# Combining AI Tools with Non-Destructive Technologies for Crop-Based Food Safety: A Comprehensive Review

**DOI:** 10.3390/foods13010011

**Published:** 2023-12-19

**Authors:** Hind Raki, Yahya Aalaila, Ayoub Taktour, Diego H. Peluffo-Ordóñez

**Affiliations:** 1 College of Computing, University Mohammed VI Polytechnic, Ben Guerir 43150, Morocco; yahya.aalaila@um6p.ma (Y.A.); peluffo.diego@um6p.ma (D.H.P.-O.); 2 Materials Sciences and Nanotechnoloy (MSN), University Mohammed VI Polytechnic, Ben Guerir 43150, Morocco; ayoub.taktour@um6p.ma

**Keywords:** chemometrics, food contaminants, food processes, machine learning, spectroscopy, sustainability

## Abstract

On a global scale, food safety and security aspects entail consideration throughout the farm-to-fork continuum, considering food’s supply chain. Generally, the agrifood system is a multiplex network of interconnected features and processes, with a hard predictive rate, where maintaining the food’s safety is an indispensable element and is part of the Sustainable Development Goals (SDGs). It has led the scientific community to develop advanced applied analytical methods, such as machine learning (ML) and deep learning (DL) techniques applied for assessing foodborne diseases. The main objective of this paper is to contribute to the development of the consensus version of ongoing research about the application of Artificial Intelligence (AI) tools in the domain of food-crop safety from an analytical point of view. Writing a comprehensive review for a more specific topic can also be challenging, especially when searching within the literature. To our knowledge, this review is the first to address this issue. This work consisted of conducting a unique and exhaustive study of the literature, using our *TriScope Keywords-based Synthesis* methodology. All available literature related to our topic was investigated according to our criteria of inclusion and exclusion. The final count of data papers was subject to deep reading and analysis to extract the necessary information to answer our research questions. Although many studies have been conducted, limited attention has been paid to outlining the applications of AI tools combined with analytical strategies for crop-based food safety specifically.

## 1. Introduction

*One Health* is a very distinct concept unifying soil, plant, and human health for a flourishing and sustainable ecosystem. This approach can also be applied on ensuring food safety and security. Ending hunger and ensuring access to safe, nutritious, and sufficient food all year round by all people are part of the Sustainable Development Goals (SDGs) to be achieved by 2030, according to the United Nations SDGs agenda, specifically SDG3 and SDG2, respectively (Good Health and Well-being, Zero Hunger). Nonetheless, multiple challenges and issues are increasingly making this mission impossible to accomplish [1]. As a matter of fact and according to statistical studies, humanity is expecting an enormous increase in the global population, reaching 9.7 billion individuals by 2025. Surely, ensuring that they all have access to safe and nutritious food becomes more challenging. The increasing demand for crop-based food in the market is due to multiple factors, mainly the propaganda of plant-based diets [2], the transition of human diets in society, and the factor of poor diversity in crop-food consumption while thousands of crops exist. These factors have impacted diversity concerning crops, and lately, scientists have been shedding light on orphan crops, which are crops not traded internationally, but would have been important for regional food security [3], hence our objective to target food of crop origins.

Concretely, it has always been a big challenge; the food system dates back several decades, with a tendency to evolve continuously, depending on its economic, social, cultural, and environmental factors, along with several external and internal variables [4]. These complex interdependent and interconnected factors impact the global agrifood system, encompassing the farm-to-fork continuum and various environmental and socioeconomic factors [5]. In this dynamic context, the integration of crop classification using deep learning emerges as a significant consideration, providing advanced tools for precise and efficient analysis within the intricate web of factors influencing the agrifood system, supported by numerous studies dedicated to the development of advanced tools [6,7]. Hence, food security, quality, and safety, now more than ever, are depending on the entire food supply chain from early production to market accessibility. A food-borne disease is referred to as food contamination, on account of the presence of hazardous contaminants that can cause human body illnesses. They are divided into three groups, namely biological, chemical, and physical contaminants, based on the pollutant and the process by which they enter the food product [8], for example, during crop cultivation, due to contaminated soil (animal manure or chemical fertilizers), as well as the water used for irrigation of produce (groundwater, recovered rainfall, surface water, or re-utilized wastewater) [9,10]. Food contamination represents a big challenge because of its large impact, being related to the whole food supply chain. In order to protect consumers from unsafe foods, standards are required to establish a monitoring system to reduce chemical and microbiological food contamination [11], including all food chain participants, such as farmers, processors, transporters, retailers, and consumers [4]. Even though there are many guidelines to follow, only a fraction of them is followed. In many cases, individuals neglect simple practices, such as proper hand-washing methods and the good use of gloves, which leads to serious food poisoning [12].

It was said that the transmission of viruses through food matrices is less likely compared to direct person-to-person contact, respiratory droplets or contaminated surfaces. The COVID-19 pandemic has forced us to think about the technological preparedness to use these smart technologies, which avoid human-to-human and human-to-food contact during food processing. Hence, there is a need for technological innovation combining analytical strategies with AI tools, taking into consideration economic and feasibility challenges [13]. Artificial intelligence (AI) is primordial for technological advances, developing computational tools to provide sustainable solutions for food security and safety [14]. There are potential applications of machine vision systems in addition to analytical strategies for more accurate and lower-cost techniques for contaminant detection in food [15].

The present work proposes a unique methodology that was developed after a careful investigation of the literature on how to conduct a high-quality comprehensive review. For the data collection, which is detailed later on, the search process is carried out in three major databases: Scopus, Web of Science, and IEEE Xplore. Notwithstanding the fewer databases implicated, the proposed approach is based on executing multiple query searches in each one, leading to an in-depth and exhaustive investigation.

The remainder of this paper is organized as follows. In Section 2, we start by investigating related and/or similar works to our topic of research. Section 3 holds the section in which research questions, search procedures, and paper selection settings are described. Section 4 presents and discusses relevant information extracted from collected papers according to our research questions. In Section 5, we highlight further details concerning gaps in research and challenges. Finally, Section 6 draws conclusions, final remarks, and our suggested future research directions.

## 2. Background

Spectroscopic measurement methods are versatile tools applied across diverse scientific domains. They play crucial roles in pharmaceutical quality control, environmental monitoring, materials science, medical diagnostics, forensic analysis, agricultural research and much more. The adaptability and precision of these methods make them invaluable for characterizing composition, structure, and properties in various scientific disciplines. Recent advances have emerged in chemical analytical strategies, depending on the variety of studied food matrices. There is a constant quest to find the most suitable technique to investigate certain aspects of a compound and ascertain its consistency or structure.

### 2.1. Spectroscopy for Food Safety and Quality

Spectroscopic methods serve as analytical tools to identify food’s composition, germs, pests, diseases, and adulteration [16]. The following listing presents the most common techniques within the literature: 1. infrared spectroscopy, 2. Raman spectroscopy, 3. nuclear magnetic resonance (NMR) spectroscopy,4. ultraviolet–visible spectroscopy (UV-vis). Infrared spectroscopy using Fourier transform infrared (FTIR) is often employed. The mid-infrared (MIR) region covers an area between 4000 and 400 cm${}^{-1}$ [17]. A soil’s composition, characteristics, and organic matter may all be found via MIR spectroscopy. Also, the diffuse reflectance infrared Fourier transform (DRIFT) method may identify the chemical characteristics of humus and soil. Moreover, attenuated total reflectance (ATR) can identify organic materials in soil [18]. Anisidine, which is produced during the oxidation of food, is frequently measured using UV-visible spectroscopy to assess the quality of oil [19]. Moreover, fluorescence is a property shared by a large number of microorganisms, including their colonies, making it simple to identify any bacteria by looking at their fluorescence spectra [20,21]. Tryptophan, riboflavin, and lumichrome are three different forms of fluorophores found in yogurt, and their presence enables fluorescence spectroscopy to assess the yogurt’s quality [22]. Honey is a substance produced by bees from floral nectar, containing phenolic chemicals that are byproducts of the phenolic acid found in the flower, and as it is packaged and transported, its qualities alter [23]. Fluorescence spectroscopy can quantify the concentration of phenolic chemicals. During processing and storage, mycotoxins and fungus are found in grains using MIR spectroscopy [24]. On the other hand, carbohydrates’ structure can change during storage, especially in the presence of water, which can be identified using Raman spectroscopy. This technique characterizes and quantifies the lipid content of foods [25]. NMR spectroscopy can monitor the ripening, drying, and adulteration of food components as well as determining the genotype responsible for a certain phenotype of the grapes used to create wine. As a result, NMR spectroscopy may offer information on mixtures of metabolites [26]. Infrared or Raman fingerprints are the outcomes of observations made on a large number of objects or samples with a wide variety of characteristics (variables, like the absorbance at various wave-numbers or wave-number shifts) from a monochromatic light source for FTIR and Raman, respectively [27]. Therefore, since the exponential rise in computing power and the capacity to gather, store, and analyze enormous volumes of data, machine learning (ML) systems can improve the potential to extrapolate information from complicated spectrum data.

### 2.2. Integrating AI Tools for Food Safety and Quality Analysis

The development of analytical strategies for food and beverage assessments is crucial for ensuring food safety and public health. Various technologies, including imaging, odor, and taste, have been developed [28]. Spectroscopic techniques, such as infrared spectroscopy and Raman spectroscopy, have proven to be rapid and nondestructive for microorganism detection [29]. Magnetic surface-enhanced Raman scattering nanoprobes show high specificity in separating and detecting multiple pathogens in complex food matrices [30]. The usage of electronic noses, coupled with data acquisition cards and classification methods, enhances success rates in ensuring the originality of saffron products [28]. The integration of more effective algorithms is essential for spectral data processing and microorganism reference database building [29].

Hyperspectral imaging (HSI) systems, including computer vision systems (CVS), are widely applied in the food industry for nonintrusive quality control [28]. HSI covers various food-processing phases, providing the ability to control the quality and safety of processed foods [31]. Challenges lie in the classification, especially for crops within the same family, and the high cost of these technologies [31]. Algorithms and chemometric methods should focus on reducing the dimensionality of data and improving computational efficiency while enhancing performance and robustness [31].

Terahertz spectroscopy techniques, coupled with machine learning tools, ensure quality and security inspection of agricultural products and food [32]. Classical methods of spectral preprocessing, such as smoothing, standard normal variate, and Fourier transformation, can be integrated into multivariate calibration steps for more efficiency [33]. In the quantification of honey adulteration, spectroscopy and hyperspectral imaging, when coupled with machine learning models and optic fiber sensors, provide fast and nondestructive detection [34].

AI development in data mining has made significant breakthroughs, particularly with the application of deep learning (DL) in the analysis of spectral data from food and agricultural products [35]. DL approaches offer a less laborious yet more precise method for this purpose. Combining infrared spectroscopy (IRS) and hyperspectral imaging (HSI) techniques with AI tools shows potential in advancing the quality evaluation of cereals, which are among the top consumed crops globally [36]. The integration of convolutional neural networks (CNNs) in the qualitative and quantitative analysis of spectra involves extracting micro- and macro-features through multiple convolution and pooling layers. DL-spectroscopic sensing techniques have demonstrated promising results in the quality evaluation of food and agro-products, encompassing identification, geographical origin detection, adulteration recognition, bruise detection, and component content prediction for crops [35].

The application of CNNs helps avoid secondary workloads, although challenges persist, such as determining optimal network scale, selecting parameters, addressing overfitting, and enhancing model interpretability—a current dilemma in AI research [33]. Table 1 highlights recent and high-quality papers that couple analytical strategies with machine learning approaches across diverse food safety and quality purposes.

## 3. Methodology

**Figure 1:**
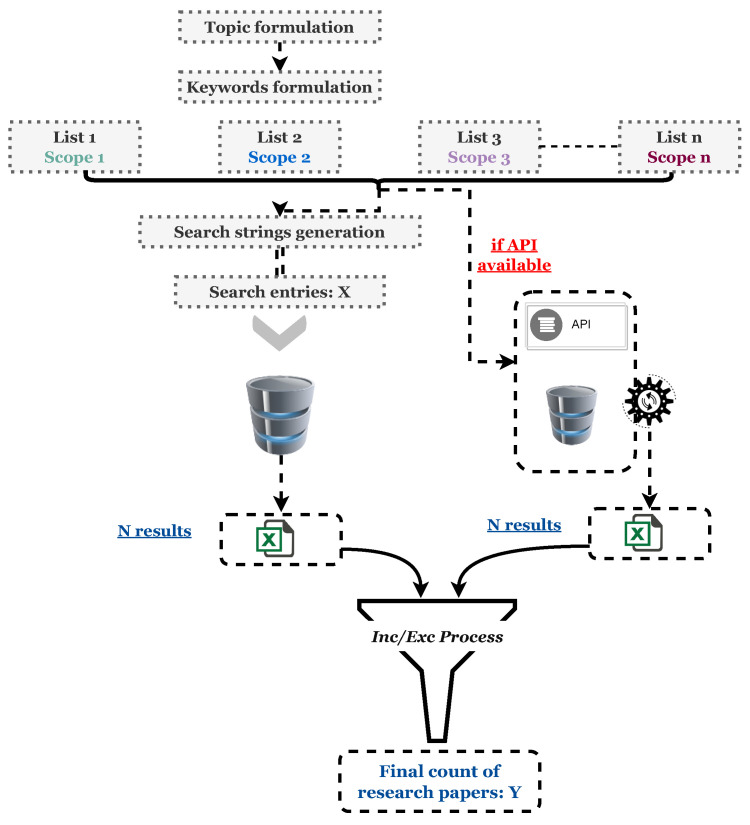
Generalized representation of our *MultiScope Keywords-based Synthesis* methodology.

Writing a quality review paper is a crucial step in one’s research project; it helps in clarifying the state of knowledge, explaining apparent contradictions, identifying needed research, and creating a consensus where none existed before [44]. In the literature, there are three major types of review papers: comprehensive (including systematic reviews), semisystematic, and integrative, depending on its purpose, research questions, search strategy, and data analysis. However, they all generally aim at resolving conceptual ambiguities by providing an integrated, synthesized overview of the current state of the art, as well as presenting research insights, existing gaps, and future research directions [45]. A good literature review must offer both depth and rigor; hence, demonstrating an appropriate strategy for selecting articles and capturing data and insights is crucial [46]. To this end, we propose our own methodology for a comprehensive review: *TriScope Keywords-based Synthesis*, which can be further generalized to a *MultiScope Keywords-based Synthesis* methodology, as represented in Figure 1. The following section describes in detail the process and methodology that we developed to execute this study, taking into consideration the guidelines of writing a review paper [45,46].

### 3.1. Topic Formulation

This study reviews the current advancements in incorporating AI tools to address challenges associated with analytical procedures and advanced chemical methods. These approaches are applied to prevent food contamination within the food supply chain. For this matter, we propose the following questions:

- What are the analytical strategies that are mainly used for crop-food safety and which techniques were dominantly incorporated?
- What are the AI-based tools that were embodied to ensure crop-food safety?
- Did these AI-based systems prove to be beneficial in research and industry? To what degree they proved to be explainable or/and interpretable?

Depending on the type of questions and the targeted scientific audience, the collected papers are analyzed in different manners, either pursuing a qualitative or quantitative analysis. In our case, and while this topic combines three different fields of research, we focused on both quantitative and qualitative analysis, in order to allow people from either fields to comprehend the general outline of this work. In order to answer these questions, an efficient and in-depth analysis of the available literature regarding the addressed topic has been generated and fully explained in the following section.

### 3.2. Study Design

**Figure 2:**
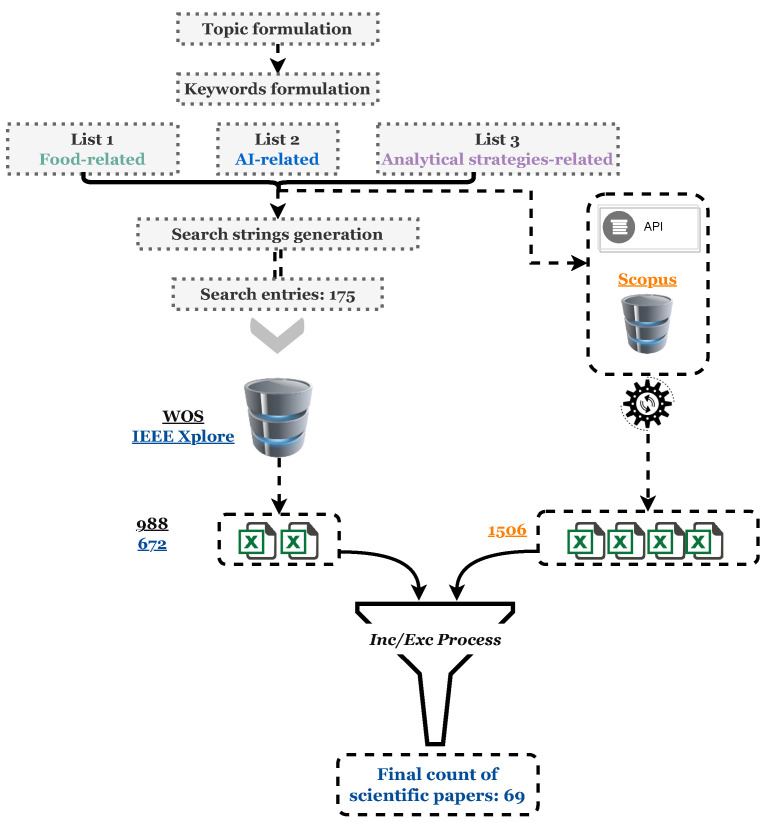
The general workflow of our developed *TriScope Keywords-based Synthesis* for conducting this comprehensive review.

Academic articles are available through a plethora of scholarly databases. Web Of Science (WOS), Scopus, and IEEE Xplore contain, combined, over 254 million records with a variety of influential academic journals, and are highly recognized by the international academic community. They cover a wide variety of topics, where many papers from different publishers are visible, and they allow access to diverse databases, with the possibility of filtering the search based on personalized search criteria. This paper combines the above three databases as the main source of articles. In order to answer our proposed questions related to our topic of research, we followed a thorough search methodology as visualized in Figure 2, considering the three above-mentioned databases for paper retrieval. The whole process of data collection, including keyword identification, search string generation, and inclusion/exclusion, was performed from October 2022 to January 2023.

**Figure 3:**
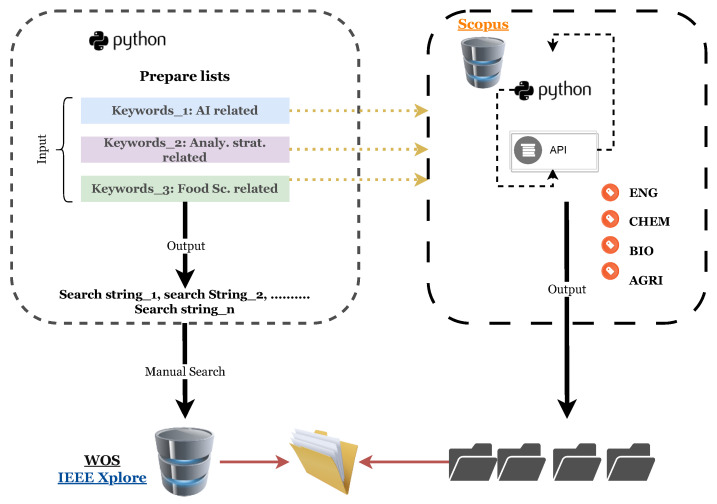
Data collection process, using API for the Scopus databse and manual searching for Web of Science and IEEE Xplore databases.

These processes were conducted using two approaches, one for the WOS and IEEE Xplore databases, which depended mainly on manual search, and an API-based approach for the Scopus database (see Figure 2 and Figure 3). Figure 2 summarizes the whole process of data collection for all three databases. All searches targeted papers of the same timeline, which is from 1 January 2000 to 30 September 2022. We aimed to explore the evolution of this issue over an extended period, allowing for a comprehensive investigation and analysis.

#### 3.2.1. WOS and IEEE Xplore Data Collection

The first step is the presearch phase. Firstly, we collected the most convenient and in-domain concepts and technical words to describe this topic by investigating the most relevant literature and executing manual preliminary searches on the Google Scholar database, in order to define our keywords. The list of keywords that were used during our search process, divided into three groups:

- Food: food, foodborne, crop, cereal, and toxin.
- Analytical Strategies: analytical strategies, biochemistry, chemical analysis, spectroscopy, omics, immunosensor, and biosensor.
- AI tools: artificial intelligence, machine learning, deep learning, neural networks, and computer vision.

These keywords were then divided into three columns, which represent the three main parts of this research: the first one for AI, the second one for analytical strategies, and the third one for food safety keywords. Secondly, we organized and ran a simple algorithm on Python, which was able to produce all possible combinations between these words from the three columns, to have as an out-put a three-word search string entry, stored in a xlxs. format file. The order of these words isn’t important in this case, and the final query string is a combination of three words from three lists of keywords corresponding to food, AI tools and chemical analytical strategies. The final count of these generated search string was 175 query string, written as follows:

$$\begin{array}{cc} Search\;String{\;}_{1} & =“Artificial\;intelligence”+“Analytical\;strategies”+“cereal”\\ Search\;String{\;}_{2} & =“Artificial\;intelligence”+“Analytical\;strategies”+“crop”\\ Search\;String{\;}_{3} & =“Artificial\;intelligence”+“Analytical\;strategies”+“food”\\ Search\;String{\;}_{4} & =“Artificial\;intelligence”+“Analytical\;strategies”+“foodborne”\\ Search\;String{\;}_{5} & =“Artificial\;intelligence”+“Analytical\;strategies”+“toxin”\\ Search\;String{\;}_{6} & =“Artificialintelligence”+“Biochemistry”+“cereal”\\ \; & \vdots \\ Search\;String{\;}_{175} & =“Neural\;networks”+“spectroscopy”+“toxin” \end{array}$$

These search queries were inserted as described previously for each search in the WOS database, but they were converted to another form using the and Boolean operator and the All Metadata filter in order to execute the manual search in IEEE Xplore, which leads to:

$$\begin{array}{c} Search\;String_{1}=\left(“All\;Metadata”:Artificial\;intelligence\right)\mathrm{AND}\\ \left(“All\;Metadata”:Analytical\;strategies)\mathrm{AND}(“All\;Metadata”:cereal\right) \end{array}$$

Along with these specific keyword combinations, we used database filters to display results based on some specific criteria, which are the timeline (2000–2022) and paper status (only published papers).

In both databases, a total of 175 searches were executed manually between the 30th of October 2022 and the 4th of November 2022 (Figure 3). The outcome of each search resulted in a number of results, ranging between 0–63 results for IEEE Xplore and 0–235 results for WOS. Each search outcome was stored as a link in a xls. format file with its according search string, sorted from the highest to the lowest, in order to eliminate search strings that did not generate any results. These links needed to be briefly inspected in a short period to avoid any research papers being added on the databases. Table 2 highlights the six search strings that provided the highest numbers of results for both databases (more than 30 results).

#### 3.2.2. Scopus Data Collection

To retrieve data from Scopus database, an open access Application Protocol Interface (API) was used in Python. Three string lists were defined, corresponding to Food, Chemical Analytical strategies and AI tools keywords as mentioned before. Subsequently, a list of four subjects was considered, containing Engineering (ENG), Chemistry (CHEM), Biology (BIO), and Agriculture (AGRI). To formulate one specific query, we looped over all possible combinations of keywords formatted in accordance to the standard Scopus advanced search. Then, we retrieved corresponding data for each query in the four considered subjects. Then, we stored the obtained metadata in four .xlsx files corresponding to the considered subjects (see Figure 3).

#### 3.2.3. Inclusion and Exclusion Process

The three databases resulted in a total number of **3166** results as raw data, which went through the same scanning process. The inclusion/exclusion (Inc/Exc) phase consisted of three major subphases, each one bases its outcome on distinguished and precise criteria of selection:

- **Inc/Exc 1:** A first selection based on the relevance of these papers to the topic, after thoroughly reading the title, key words, and abstract sections, respectively. The number of papers included were **30**, **166**, and **242** for IEEE Xplore, WOS, and Scopus databases, respectively.
- **Inc/Exc 2:** A further evaluation of these collected papers was conducted based on their original language and availability online, also removing duplicates, which ended with a total of **109** papers included.
- **Inc/Exc 3:** A full reading process was thoroughly executed to decide which of these papers are the most relevant to our topic of research, leaving **69** papers.

**Figure 4:**
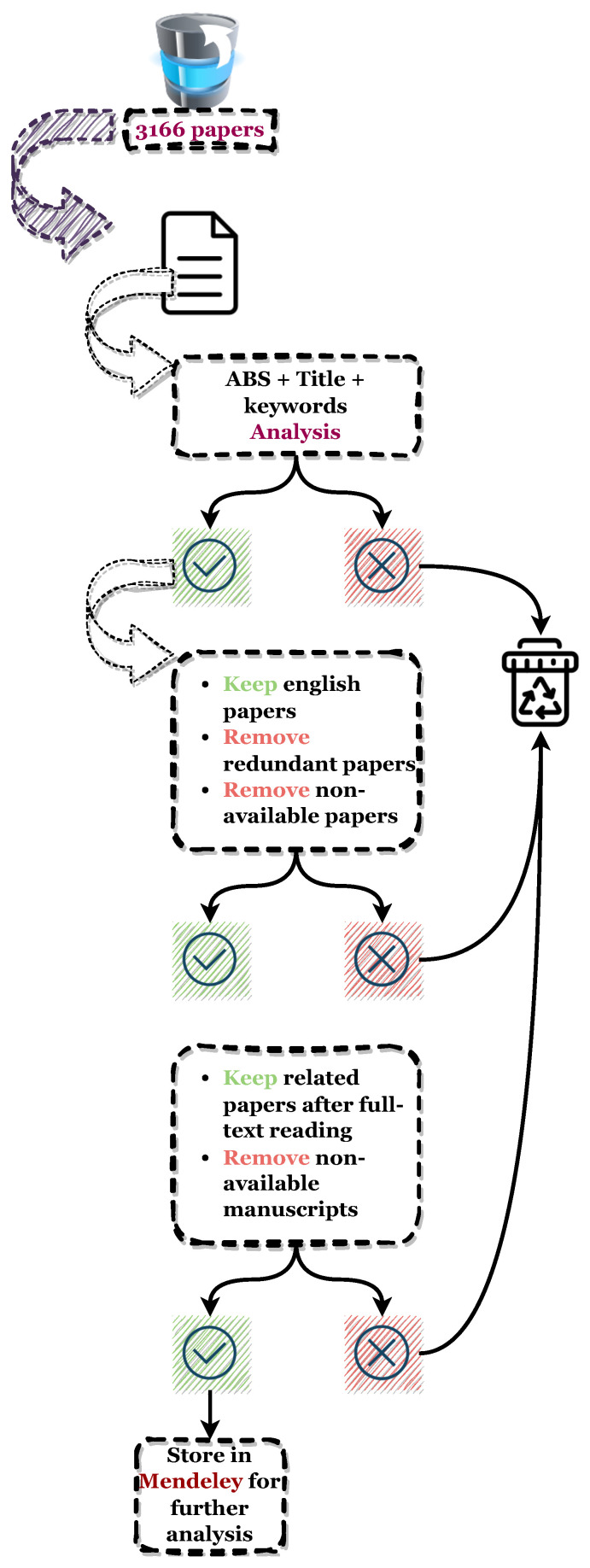
Diagram illustrating the inclusion–exclusion process and criteria of selection.

The final count was 69 research papers that have met all the search criteria, and were further organized in Mendeley https://www.mendeley.com. This software will serve as a tool for storing and analyzing these papers. Figure 4 summarizes in details the process of Inc/Exc phase.

## 4. Data Description and Analysis

**Figure 5:**
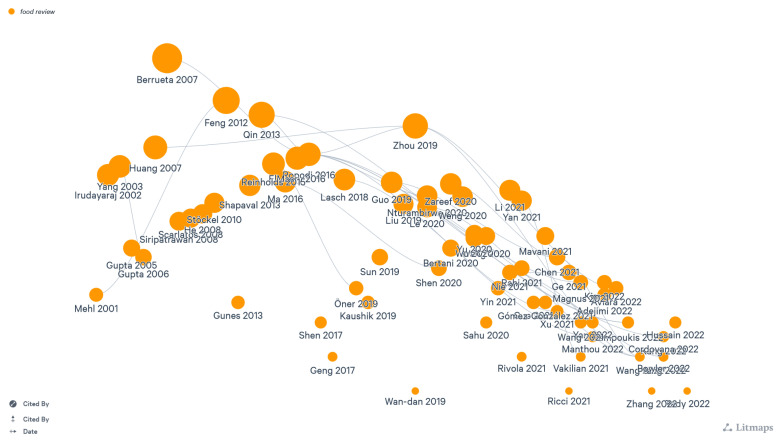
A map of all our collected papers. Each node represents a paper with its first author and year of publication. The size of a node represents the number of citations. Please refer to Table 3 for more details.

As discussed in Section 3, our methodology output is 69 papers, including articles and reviews, as detailed in Table 3. Figure 5 also showcases a map of these papers with possible connections and links, highlighting first authors and the according year of publication, as well as numbers of citations for each one. This map was produced using the free online tool ConnectedPapers https://www.connectedpapers.com/, after manually uploading our collected papers. The following data results representation will focus on quantity and quality analysis, as set forth in the upcoming parts.

### 4.1. Quantitative Analysis

**Figure 6:**
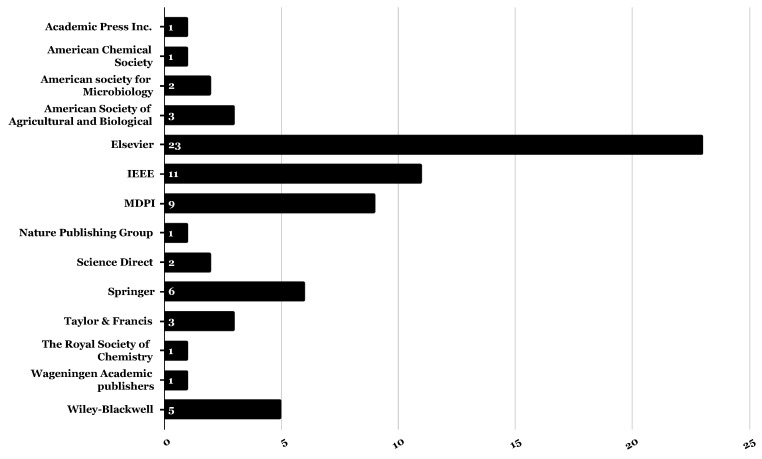
Count of published papers per publisher name.

Although we explored a larger period of time, from 1 January 2000 to 30 September 2022, most of our collected papers were between 2019 and 2022. As shown in Figure 6, these papers were generally published in 14 different publishers, which are well classified globally and highly recognised.

**Figure 7:**
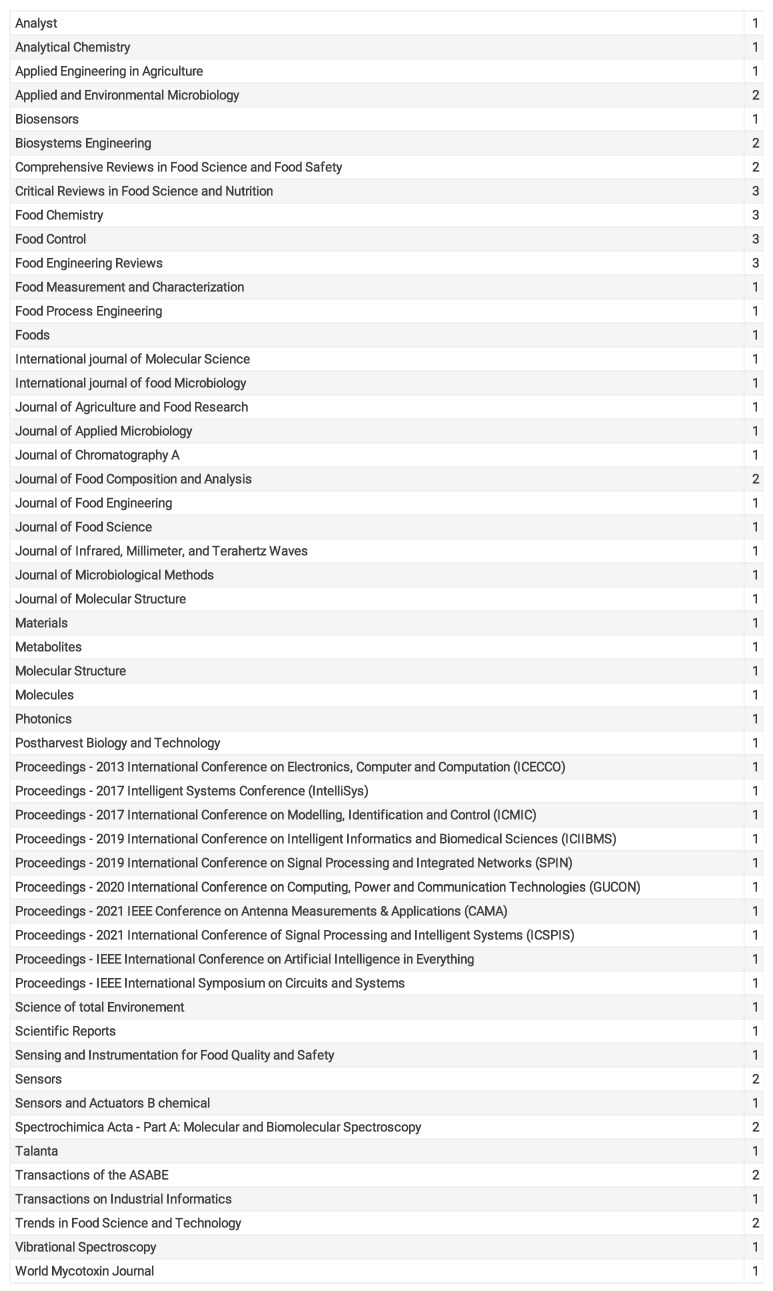
A table chart depicting the number of papers published per journal.

Most of the journals are highly ranked, with a quartile of 1 (Q1), which are divided as article papers and review papers; some of these papers, mainly those published by IEEE, are conference papers, as in Figure 7.

**Figure 8:**
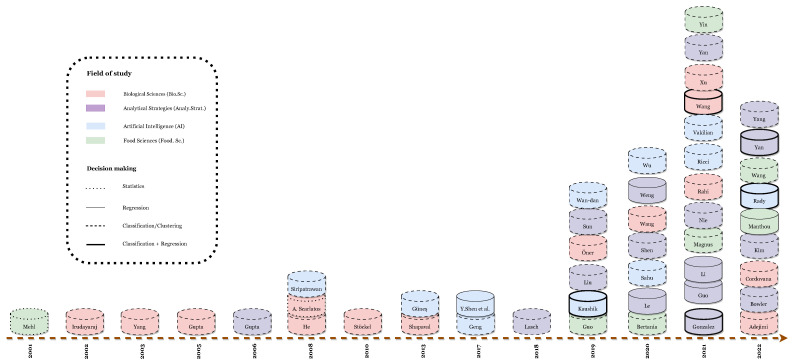
A diagram representing the chronological (from 2001 to 2022) distribution of the resulting articles. Each data-point represents an article, the colors are according to which field of study, and the lines are related to the decision-making objective Mehl, 2001 [47]; Irudayaraj, 2002 [48]; Yang, 2003 [49]; Gupta, 2005 [50]; Gupta, 2006 [51]; He, 2008 [55]; A. Scarlatos, 2008 [54]; Siripatrawan, 2008 [56]; Stöckel, 2010 [57]; Günes, 2013 [60]; Shapaval, 2013 [59]; Geng, 2017 [66]; Y.Shen, 2017 [67]; Lasch, 2018 [68]; Guo, 2019 [72]; Kaushik, 2019 [69]; Liu, 2019 [74]; Öner, 2019 [71]; Sun, 2019 [75]; Wan-dan, 2019 [73]; Bertania, 2020 [85]; Le, 2020 [77]; Sahu, 2020 [80]; Shen, 2020 [84]; Wange, 2020 [78]; Weng, 2020 [79]; Wu, 2020 [83]; Gonzalez, 2021 [93]; Guo, 2021 [94]; Li, 2021 [95]; Magnus, 2021 [91]; Nie, 2021 [101]; Rahi, 2021 [86]; Ricci, 2021 [97]; Vakilian, 2021 [96]; Wang, 2021 [92]; Xu, 2021 [90]; Yan, 2021 [99]; Yin, 2021 [98]; Adejimi, 2022 [109]; Bowler, 2022 [107]; Cordovana, 2022 [110]; Kim, 2022 [108]; Manthou, 2022 [115]; Rady, 2022 [106]; Wang, 2022 [111]; Yan, 2022 [104]; Yang, 2022 [102].

On the other hand, Figure 8 highlights the distribution of article papers only, in a color-coded format; each color refers to a field of study. Although our keywords were mainly extracted from three different fields of research, after the paper collection, another distribution emerged. These fields are Artificial Intelligence (including computer vision, machine learning, etc.), Analytical Strategies, Biological Sciences, and Food Sciences.

We will discuss these papers’ contents while trying to answer our research questions (RQs), which are considered as the qualitative analysis of our results. Firstly, we divide the discussion into two parts, one for review papers and all related types, and the second one for article papers with experiments and applications. Then, the outcome from discussing these review papers will help as a reference while discussing the articles.

### 4.2. Qualitative Analysis

**Figure 9:**
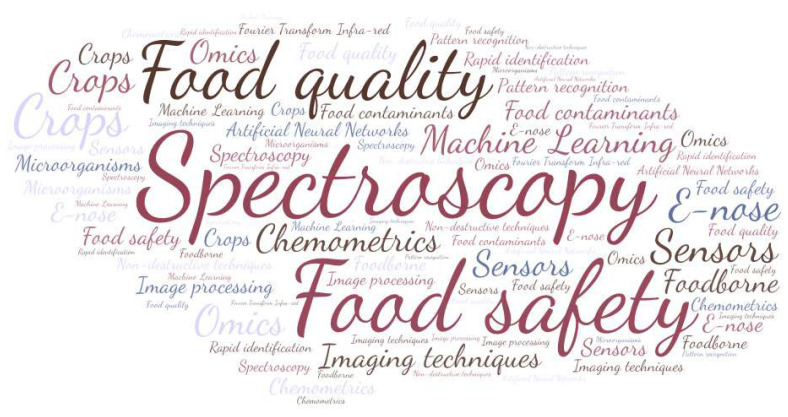
A map highlighting subject-related words and concepts that were strongly present within the literature, such as Spectroscopy (Graphical design generated using free-online tool Wordart https://wordart.com/create, accessed on 23 August 2023).

Over the last decade, there has been a rapid development of artificial intelligence (AI) tools for the nondestructive evaluation of food and agricultural products. However, a key aspect of the emerging AI tools is the preprocessing phase, where acquired data are adequately prepared for further analysis and decision making using, generally, machine learning models. Through our analysis process of our data papers, new keywords and concepts emerged, for a diversity of techniques and technologies, regarding analytical strategies where spectroscopy and chemometrics were the most used in articles and discussed in review papers (see Figure 9). The commonality among these collected papers is their focus on exploring innovative approaches and technologies applied to food sciences. Their aim is to solve challenges related to food safety, quality assessment, authenticity verification, and inspection processes. In the following sections, we will discuss articles and review papers separately, following the three RQs defined in the Methodology section.

### 4.3. Review Papers

The review papers report recent advances regarding the application of various AI technologies and methodologies in the field of food sciences. These papers discuss the use of different techniques in AI, such as artificial neural networks (ANNs), computer vision, data mining, deep learning, machine learning, and statistical modeling combined with recent analytical strategies, such as hyperspectral imaging, chemometrics, nondestructive analytical techniques, THz spectral imaging, bioimpedance and spectroscopic methods for food analysis, quality assessment, safety inspection, authenticity determination, and defect detection. Assessments of food products are divided into two pillars: safety and quality parameters, and since our concern is on safety, we focused on extracting information related to this matter, specifically for crop-based food.

#### 4.3.1. Analytical Strategies and AI as Nondestructive Tools for Crop-Food Safety

These review papers are commonly discussing the usage of analytical techniques and consider them as revolutionary, mostly for being nondestructive Techniques in food analysis, where the key word “nondestructive” was stated plainly in these works [62,63,64,76,82,88,89,100,112]. These techniques allow for the assessment of crop-based food safety without structural damage. The overall focus of these papers focuses on both aspects of food analysis: safety and quality [52,61,70,112]. Nonetheless, we will inspect only technologies and methodologies that have been implemented in food safety analysis, thus detecting contaminants, assessing microbial risks, determining the presence of pathogens, and evaluating their safety and readiness to be manufactured, stored, transported, or directly consumed. Many examples emerged, such as reviewing methods for determination of contaminants in spices and herbs [62], such as biological contaminants [113,114], caused by foodborne pathogenic microorganisms. These samples are subject to analytical analysis, which then, depending on the objective of the study and which aspect of food safety to tackle, will produce data.

The most referred-to analytical strategies in solving this matter were spectral imaging (SI) technologies [61,113], and spectroscopy-based technologies [52,61,63,64,76,81,82,88,89,100,113,114].

Different techniques have emerged in spectral imaging, like hyperspectral imaging (HSI) [58,61,65,70,82,103,112], for example, near-infrared hyperspectral imaging [112], as well as multispectral imaging [58,61,64,65,113] and THz spectral imaging [88]. In spectroscopy, we list ultraviolet-visible spectroscopy (UV-vis), near-infrared spectroscopy, Fourier transform infrared (FT-IR) spectroscopy, Raman spectroscopy, THz spectroscopy, THz time-domain spectroscopy(THz-TDS), laser-induced breakdown spectroscopy (LIBS), surface-enhanced Raman spectroscopy sensors (SERS), nanoenzymes, and modified chromatographic techniques that were mostly coupled with AI tools [76,81,88,100,112,113,114]. On the other hand, electroanalytical methods have risen to mimic the human senses by using sensor arrays and pattern recognition systems, known as E-nose, E-tongue, and E-eye [81]. They are often combined with other spectral techniques for real-time detection and higher accuracy results, such as near-infrared spectroscopy (NIRS) [87]. The E-nose is destined to emulate the human olfactory system, hence avoiding exposure to dangerous chemical and biological hazards due to inhaling or skin leisure [82]. Many applications have been studied, like evaluating shelf life by detecting grain off-odor due to microbial spoilage for wheat and barley-based foods [52]. Furthermore, electrochemical impedance spectroscopy (EIS) has been receiving more attention research lately, especially when applied on biological tissues, such as crop-based food membranes. EIS or bioimpedance technology is optimal for surface contaminant detection, with its capacity to detect defects [89].

Several papers discuss the importance of data analysis and processing for food safety. Chemometrics [81,100], machine learning [87,88], computer vision [63,113], and data mining [64,70] are applied to extract meaningful information, detect patterns, and make predictions based on the collected data. Machine learning techniques are summarized in Table 4, with the most common techniques gathered from some relevant review papers related to crop-food safety.

The best description of the purpose of chemometrics was in a recent work by Yu et al. [81], quoting exactly “Chemometrics, the art of extracting chemically relevant information from data produced in chemical experiments”. We can also distinguish between chemometrics and sensometrics, respectively, studying the relationship between the measured chemical parameters and the state of the object by statistical or mathematical methods or studying the link between sensory parameters and the internal characteristics of the object via similar approaches as the first one [70]. Spectra processing and model establishment are two main aspects of chemometrics; each contains multiple analysis methods [100]. These systems that are based on sensors equipped with chemosensitive materials for molecular recognition [113] can also be joined with other techniques for data analysis in electronic sensing [81]. Chemometrics were usually incorporated within data analysis in electronic, E-nose, E-tongue, and E-eye [100] and within spectra processing and model establishment when combining AI tools with spectroscopic techniques. There is application potential of deep learning in chemometrics and sensometrics toward food [70], for example, real-time toxins quantification and detection in cereals by combining nondestructive techniques with chemometrics, such as spectroscopy-based techniques [100]. They were also joined when applied in condiment analysis methods for the determination of contaminants in spices and herbs [62]. Final data acquired by HSI systems were described as “hypercube” for their 3D characteristics: two for spatial coordinates and one for spectral values [58].

#### 4.3.2. Interdisciplinary Approaches in Crop-Food Safety

The review papers often involve interdisciplinary approaches, combining knowledge and techniques from fields such as food science, computer science, spectroscopy, image analysis, and statistical modeling. These collaborations aim to leverage the strengths of different disciplines to address complex challenges in food analysis and safety. Firstly, image correction before implementing chemometric algorithms is necessary to reduce the noise to enhance the signal-to-noise ratio by either spectral or image analysis, Secondly, data preparation and preprocessing use chemometric methods, such as principal component analysis (PCA) and partial least square regression (PLSR), to be fed for model application [58]. Microbiological applications, for mycotoxin detection, for example, include combining nondestructive methods with statistical methods such as PLS at different stages of the food supply chain; harvesting and transporting, due to many climate conditions and agricultural processes [62]; and the detection of the total aflatoxin content using fluorescence fingerprints (FF) in combination with PLSR [62]. FT-NIR data were often analyzed using the PLS regression with various preprocessing techniques, such as straight-line subtraction (SLS), constant offset elimination(COE), and minimum–maximum normalization (MMN). The classification of hyperspectral images of possibly contaminated chili was proposed by using PCA and SANN as a classifier [62]. Supervised ML approaches (classification and regression) are used for the detection of endogenous component content, pesticide residue, microplastic, and heavy metal contamination in crops [103]. THz-TDS can be combined with SVMs for classification purposes, scoring good results [81]. However, this work can be criticized for mentioning various spectral dimensionality reduction techniques for feature extraction and selection, like LLE, LE, ISOMAP, and MDS in addition to SNE and t-SNE, whereas those techniques are not mentioned in the literature in any shape or form [103]. PCR, PLS, and MLR are best to check the linearity, while qualitative techniques are best for non-linearity detection, such as partial residual plot (PRP), residual plot (RP), e-PC (AVP/PaRP), and Mallows’ augmented partial residual plot (APaRP). On the other hand, quantitative techniques are mostly referred to for statistical methods such as ANOVA for lack of fit (LOF) for univariate calibration mode, or the most basic tests to assess serial correlation: the Durbin–Watson test and run test [103]. The fuzzy logic technique is used in the food industry, in food modeling, control, and classification, and in addressing food-related problems by managing human reasoning in linguistic terms. FL has been proven to successfully maintain the quality of the foods, and it acts as a prediction tool and control system for food production processes [87]. Deep learning has been introduced into the food field by analyzing RGB images and spectra images of food, as the data analysis tool to solve the problems and challenges in the food domain. Food recognition and classification, such as CNN and image analysis, has been the most commonly used pattern in food recognition and classification. Numerous popular CNN architectures for image processing were cited, including AlexNet, the visual geometry group network (VGG), GoogLeNet, and the residual neural network (ResNet), as well as the usage of hybrid models, such as radial basis function (RBF) kernel-based SVM with ResNet-152, coupling fine-tuned AlexNet with a binary SVM classifier [70].

#### 4.3.3. Analytical Strategies and Recent Technologies for Food Safety

Some papers highlight the use of automation and advanced technologies to streamline food analysis processes, improve efficiency, and enable rapid assessment. This includes the integration of computer vision, machine learning, and artificial intelligence techniques for automated defect detection and quality evaluation. Table 4 highlights some relevant reviews reporting chemometrics and ML applications for food safety and quality.

Analytical strategies deploying AI tools can be described as knowledge-based systems with computer programs using knowledge from different sources for food safety problems, such as expert systems, knowledge-based artificial intelligence, and knowledge-based engineering [87]. Integrating various sensors with AI-based methods has been increasing in food industries over the past few years, for example, E-nose-based systems to detect defects and contamination in crop foods. The classification and differentiation of different fruits have also been determined by using E-nose. CVS usage can be a handful for image processing and pattern recognition combined with near-infrared spectroscopy (NIRS) in the food industry for accurate and precise results [87]. Combining ANNs-sensors in real-time applications, like E-nose and E-tongue for real-time detection, scores faster and higher accuracy results [87]. Fecal contamination on fruits and vegetables—apples, for example—using reflectance-fluorescence, is coupled with band ratio, threshold, separation algorithms, and PCA. Defects like bruises and lesions are considered symptoms of possible contamination if caused by a biological agent. Hyperspectral imaging is then used to inspect defects coupled with many tools, such as PLSDA, ANN, LDA, and PCA [58].

Overall, the common points among these review papers revolve around the application of emerging technologies, nondestructive techniques, data analysis, food safety, quality assessment, automation, and interdisciplinary approaches within the field of food science.

### 4.4. Article Papers

As depicted in Figure 8, the obtained 49 research papers focus on the identification, and in some cases quantification, of food-borne pathogens, be they bacteria, fungi, or yeast. The employed machine learning (ML) workflow mostly consists of two major steps, namely *data preparation*, including data acquisition, data cleaning, and data transformation; and *decision making* including, tuning, training, and testing of one or multiple machine learning models.

The results are best categorized by how data were gathered. However, in most cases, data acquisition stems from a chemical analytical approach, mostly spectroscopy-based approaches, resulting in *structured data* in the form of numerical values or *unstructured data* in the form of images. Thus, the results are reported following the taxonomy outlined in Section 2.1. Each subsection that follows gathers the research papers that use the specific spectroscopic approach, and its variants if any, combined with various ML methods for food safety purposes.

#### 4.4.1. Raman Spectroscopy

In earlier studies, Raman spectroscopy was used in combination with classical machine learning approaches to enhance food pathogen detection. In [57], principal component analysis (PCA) was applied in combination with linear discriminate analysis (LDA), support vector machines (SVM), and a simple artificial neural network (ANN) architecture for classification of *Bacillus anthracis* endospores and their products. Also, Raman Spectroscopy spectra were preprocessed using a first derivative (D1) and Savitzky–Golay smoothing function and second (2)-order polynomial to enhance the spectra’s resolutions and eliminate baseline and linear slope effects, respectively [72]. The resulting signal was used as input for partial least squares (PLS), synergy interval partial least squares (siPLS), and ant colony optimization-siPLS (ACO-siPLS) to quantify the level of maize contamination by zearalenone. On the other hand, after applying the Savitzky–Golay smoothing function on the spectral information, a stacking approach combined with K-nearest neighbors (KNN) and support vector machines (SVM) was proposed in [116] to distinguish between the *Escherichia coli* and *Brucellasuis* vaccine. Later studies investigated the use of complex ANN-based models to enhance classification and quantification performances. In [78], a simple PCA approach was used to categorize three genera (Arcobacter, Campylobacter, and Helicobacter), then a one-dimensional convolution neural network (1D-CNN) and a fully connected artificial neural network (ANN) were used to classify 18 of Arcobacter species and quantify their ratio in the bacterial mixture [78]. In another study, raw Raman spectroscopy was used as an input to the fully connected ANN, genetic algorithm-ANN (GA-ANN), and particle swarm optimization-ANN (PSO-ANN) to distinguish between 12 strains of five bacteria genera, including (*Escherichia*, *Listeria*, *Vibrio*, *Shigella*, and *Salmonella*) [96].

In addition, in the cases where faster sampling, higher resolution, and better signal-to-noise ration are needed, Fourier transform (FT)-Raman is often used with promising results. In an older work, ref. [49] proposed a first attempt to use FT-Raman for a nondestructive characterization and differentiation of six different microorganisms, including the pathogen *Escherichia coli* on whole apples. PCA and canonical variate (CV) plot and cluster the data in two-dimensional scatter-plot.

Furthermore, a more sensitive version of Raman spectroscopy, namely surface-enhanced Raman spectroscopy (SERS), can be used in cases where sensitivity, selectivity, and rapid analysis are needed. In an older work [55], SERS was used to detect and discriminate among five Bacillus spores (*B. cereus*, *B. cereus*, *B. cereus*, *B. subtilis*, and *B. stearothermophilus*). Standard data preparation techniques were conducted, such as normalization, binning, smoothing, and second-derivative transformation, before using PCA and hierarchical cluster analysis (HCA) to cluster the data into three groups. However, a recent comparative study [79] demonstrates the benefit of using deep learning models such as CNNs, fully convolutional networks (FCN), and principal component analysis networks (PCANet) to determine their abilities to measure pirimiphos-methyl in wheat extract in the two input forms of one-dimensional vector or two-dimensional matrix, as opposed to classical ML methods such as random forests (RF), KNN, and SVM.

On the other hand, single-cell Raman spectroscopy (SCRS) has also been used in a recent work [99] to discriminate between 23 common strains from seven genera of food-borne bacteria (*Escherichia*, *Listeria*, *Staphylococcus*, *Cronobacter*, *Vibrio*, *Shigella*, and *Salmonella*) at the single cell level. Kernel PCA (KPCA) was used for nonlinear feature extraction, followed by a decision tree (DT) algorithm, with promising results.

In addition, low-resolution Raman spectroscopy (LRRS) has also been used in combination with SVM discriminate analysis to rapidly identify harmful cyanobacterial species and quantify their presence, demonstrating the potential of LRRS technology for the real-time detection of contaminant species within microalgal bioreactors.

#### 4.4.2. Visible and Near-Infrared Spectroscopy

Visibe/near-infrared spectroscopy (Vis/NIR) is of valuable usage for food safety, especially when a broad spectral range analysis is needed. It has been used in combination with LDA and partial least square regression (PLSR) for online detection of *Aspergillus* and *Fusarium* contamination in stored maize [86]. LDA was used for classification results of maize samples according to species of infected fungal strain and infection level. PLSR was used for the respective determination of colony counts. Also, in [84], Vis/NIR was considered with PCA in combination with various data preprocessing techniques, such as moving average (MA), multiplicative scatter correction (MSC), standard normal variate (SNV), first derivative (D1), second derivative (D2), and Savitzky–Golay (SG), and their combinations were considered based on PCA results. The prepared data were the input to the PLS-DA algorithm, as well as three of the variable selection-based variants (iPLSDA, GA-PLSDA, and VIP-PLSDA), to detect contaminants in Persian leek.

On the other hand, recent studies explored the use of near-infrared spectroscopy (NIR) coupled with computer vision in food safety applications. In [106], they used low-cost NIR sensors (NIRONE S2 and S2.5) to acquire spectra with wavelengths 1550–1950 nm and 2000–2450 nm. Standard preprocessing approaches were applied, such as autoscaling, mean centering, Savitzky–Golay, standard normal variate (SNV), and first and second derivatives. The objective is garlic powder contamination, especially allergen contamination such as peanut powder. Thus, five classification models were used (KNN, SVM, LDA, PLS-DA, and DT), to first detect the presence of peanut powder in garlic powder, then identify whether the presence was high (2–20%) or low (0.01–1%). In the latter two cases, a PLSR-based regression model was developed to quantify the presence concentration. Similarly, a deep learning-based approach was proposed in [107] for the in-line allergen classification of agrifood powders, combining domain-adversarial neural networks (DANN) and semisupervised generative adversarial neural networks (SGANN). In addition, two DL approaches were proposed in [98,111], namely a two-dimensional Markov transition field-CNN (2D-MTF-CNN) and a modified version of FCN (U-net) to monitor the aflatoxin B1 (AFB1) content in maize and identify food foreign contaminants (metallic iron, polypropylene plastic, and hair) on the surface of bread, respectively.

NIR spectroscopy in the diffuse reflectance mode has been used [67] for soluble solid content and total acid content analysis of fruits. After reducing the dimension of the acquired data to the leading three principal components, the backpropagation neural network (BPNN) and generalized regression neural network (GRNN) were proposed and compared in predicting the values of SSC and TAC in three cultivars of pears.

Furthermore, earlier works [50,51] investigate the usage of Fourier transform infrared spectroscopy (FTIR) coupled with complex ANN methods to identify five pathogenic bacteria (*Enterococcus faecium*, *Salmonella* Enteritidis, *Bacillus cereus*, *Yersinia enterocolitis*, and *Shigella*) [50], as well as to quantify four food pathogens (*E. coli O26*, *Salmonella* Typhimurium, *Yersinia enterocolitica*, and *Shigella boydii*) [51]. In both works, probabilistic neural networks (PNNs) were developed and tested. FTIR has been used for aflatoxin contamination detection in figs [60]. The forward feature selection (FFS) method was used to reduce features in spectra space, followed by three linear classifiers, namely the linear discriminant classifier (LDC), logarithmic linear classifier (LOGLC), and quadratic discriminant classifier (QDC). Also, it was coupled with smoothing and standard normal variate (SNV) for noise removal, followed by SVM and PLSDA to characterize and differentiate between Bacillus subtilis and Escherichia coli cell suspensions in food spoilage context [90]. The authors of [71] have also showed the benefits of using FTIR combined with five classification approaches, namely adaptive boosting (AdaBoost), random forests (RF), SVM, and multilayer perceptrons (MLPs) for the automated classification of contaminated maize. In a latter study [110], the FTIR spectroscopy-based IR biotyper system was used to successfully classifying a total of 958 characterized Salmonella enterica isolates (25 serogroups and 138 serovars). PCA and LDA were applied for visualization porpuses, followed by SVM and ANNs for classification. Also, an earlier attempt to combine FTIR photoacoustic spectroscopy (FTIR-PAS) with unsupervised analysis (PCA and canonical variate analysis) was explored in [48] to identify various bacteria (*Lactobacillus casei*, *Bacillus cereus*, and *Escherichia coli*), fungi (*Aspergillus niger* and *Fusarium verticilliodes*) and yeast (*Saccharomyces cerevisiae*) on apple surface, with promising results. However, to the knowledge of the authors, no subsequent research was conducted using this technique.

#### 4.4.3. Time-Domain Spectroscopy (THz–TDS)

The time-domain spectroscopy (THz–TDS) system can also be used in conjunction with ML approaches for food safety. Due to its spectral range, sensitivity, and chemical specificity, it is an appealing alternative for data acquisition. In [102], they proposed a novel metamaterial sensor to analyze multiresonance dips in spectra obtained using time-domain spectroscopy (THz–TDS) coupled with mean shift (MS) to investigate the presence of Carbendazim’s residue in crops. Also, soybean oil contamination by *aflatoxin B1* (AFB1) was investigated using the same spectroscopic method [74]. Student stochastic neighborhood embedding (t-SNE) dimensionality reduction (DR) methods were applied, followed by the backpropagation neural network (BPNN), least squares SVM (LS-SVM), and RF for classification purposes. As opposed to identification and classification problems, time-domain spectroscopy (THz–TDS) systems were used in tandem multiple linear regressions (MLR), PLS, and LS-SVM for measuring benzoic acid (BA) additives in wheat flour [75]. In a recent study, [104] used TPS systems with singular value decomposition (SVD), non-negative matrix factorization (NMF), self-modeling mixture analysis (SMMA), and support vector machines for regression (SVR) as preventative measures, that is, to identify and quantify the right components of preservatives (sorbic acid, potassium sorbate, and sodium benzoate) to precisely enhance the antimicrobial effect for merchandise safety.

#### 4.4.4. Fluorescence Spectroscopy

Fluorescence spectroscopy, which focuses on the measurement of the emitted light (fluorescence) from a sample after it has been excited by an external energy source, can also be used to acquire data for food contamination analysis. A recent work [85], presents a methodology for the analysis of fluorescence spectra of slurred almonds under 375 nm wavelength excitation for optical detection of *Aflatoxins B* in grained almonds. This application is different than conventional classification frameworks in the sense that labels of contaminated or not contaminated are generated based on multiple threshold values; thus, a single sample could have a vector of labels corresponding to each threshold value. This is referred to as multiexpert learning [117]. In this context, support vector machines (SVM) were used with a majority vote. On the other hand, other techniques of analyzing materials have been used, particularly based on the interaction of microwaves with samples. Unlike spectroscopy, microwave sensing systems operate in the microwave-frequency range, typically from hundreds of MHz to tens of GHz. Recently, a microwave sensing approach coupled with ANNs for food safety was proposed in [97], exploiting the dielectric contrast between the potential intrusion and the surrounding matter of packaged food. In addition, nuclear magnetic resonance (NMR) spectroscopy, which is a prominent analytical approach, was applied in a recent work [92] to distinguish between pathogenic and nonpathogenic bacteria, using PCA and ANNs.

#### 4.4.5. Nuclear Magnetic Resonance (NMR)

Nuclear magnetic resonance (NMR) can be of great importance in the food safety context due to its ability to provide detailed molecular information in a nondestructive manner. However, compared to the above categories, NMR is somewhat less used in the literature. Only one study [92] explored the use of NMR spectra in combination with artificial neural networks to distinguish between 10 different microbial strains such as *Bacillus*, *Salmonella*, and *Yersinia*, among others in food based on metabolite profiles. For this type of data, prior preparation is necessary, like scaling spectra to the internal concentration standard (TSP), removing noise bins, standardization, and PCA for visualization. The proposed ANN model, with hidden layers of 800 neurons, ReLu functions, and two dense layers, was compared with two classical machine learning algorithms (RF and SVM), with slight differences in performance.

#### 4.4.6. Hyperspectral Imaging

Optical spectroscopic techniques have been recently exploited for food safety as an advanced promising tool. Due to the enhanced spectral resolution and improved spacial information, hyperspectral imaging, combined with AI tools, is increasingly appealing for food safety applications. The first application was as early as 2002 [47], where hyperspectral imaging was used to characterize spectral features, and multispectral imaging combined with PCA was used for the detection of defects on three apple cultivars, namely Golden Delicious, Red Delicious, and Gala. Recently, an ANN-based method using FTIR-hyperspectral data was proposed in 2018 for the rapid and cost-effective diagnosis of pathogenic bacteria [68]. Also, in [83], active thermography and an infrared camera were used to capture thermal images for analysis using a multiview learning-based autoencoder (MVAE) for defect detection.

On the other hand, a study used visible and NIR (VNIR) hyperspectral imaging to determine the presence of viral particles in a fluid suspension as well as on a surface upon complete evaporation of its water content [93]. In contrast with the previous studies, which proved the effectiveness of using HSI to detect the presence of fungi or bacteria, detecting the presence of viruses, which are two orders of magnitude below fungi and bacteria, can be crucial to prevent the spread of plant diseases. The acquired VNIR-HSI data were used in combination with PLSDR and FFNN to detect the presence of viruses in addition to the analysis of averaged spectra for quantifcation of the viral load [93].

#### 4.4.7. Comparative Studies

In the light of recent developments and applications of machine learning in food safety, comparative studies are of great importance to understand and assess the viability of the techniques. In [108], a thorough comparative study was conducted using four different hyperspectral imaging techniques (fluorescence, VNIR, SWIR and Raman) combined with five classification approaches (LDA, LSVM, QDA, and QSVM), in addition to four simple preprocessing techniques. Also, the study in [115] provides a comparative assessment of spectroscopy-based techniques and machine learning approaches for evaluating the microbiological spoilage of ready-to-eat leafy vegetables (baby spinach and rocket). In this study, Fourier-transform infrared (FTIR), near-infrared (NIR), visible (VIS) spectroscopy, and multispectral imaging (MSI) were used, with only two regression algorithms, namely SVR and PLSR.

#### 4.4.8. Electroanalytical Methods

The data used in [80] were gathered using the daily inspection of food safety obtained from the Analysis and Testing Institute of one province in China, which includes manufacturer information, origin information, product information, production dates, inspection items, and inspection results, among others. The aim of this study is to first build an AHP-ELM model to distinguish between three types of food safety risks, namely heavy metal pollution, chemical contaminant pollution, and pathogenic bacteria pollution, then construct an AHP-ELM-based model for food safety warning. In an earlier study [54], microelectrode arrays (MEAs) obtained from Multichannel Systems GmBH were used to measure the electrical activity of cortical networks grown in a controlled environment (in vitro). The MEAs measure the electrical spikes of the lyophilized botulism toxin (BoNT A) group for further statistical analysis, in contrast with a controlled group without toxins. Furthermore, the use of cortical networks as a biosensor for botulinum toxin offers several advantages. First, the networks can be grown in a controlled environment, allowing for reproducible experiments. Second, the networks can be easily manipulated and modified to study different aspects of toxin detection. Third, the electrical signals from the network can be recorded noninvasively, minimizing the potential harm to the tissue. This approach provides a promising tool for potential applications in toxin detection in food safety research.

On the other hand, few studies explored the potential for a real-time monitoring sensing system to provide early means to assess food safety [69,80]. A brief study [69] proposed a real-time environment monitoring sensing system to monitor stored grain’s condition and potentially increase its shelf-life. First, sensors record the values of temperature, moisture content, and $CO_{2}$, among others, and store the data in an Ardinuo data logger periodically. Consequently, the stored data are communicated to a web server using WI-FI. Second, preprocessing techniques are applied in the microprocessor to reduce the inevitable noise generated by the different environment sensors. This is due to long-term drifts, temporary electrical errors, and the effects of nearby sensors. Finally, a classification approach can be used to distinguish between four classes, namely no spoilage, early spoilage, severe spoilage, and early insect infestation. A more recent study [80] proposed an edge IoT and machine learning-based approach for food quality monitoring systems to avoid food waste, which reaches 50% for root crops, fruits, and vegetables. First, three sensors are in place to gather environmental data, namely gas, temperature, and humidity sensors, after which any missing entry is replaced with the median value of the feature. Then, four classification algorithms are considered to categorize the results into three classes (fresh, semifresh, and spoiled), namely linear SVM, RBF-SVM, logistic regression, and random forests (RF). To ensure real-time monitoring, the results are communicated to the user through an Android application.

## 5. Research Gaps and Challenges

Spectral imaging, along with spectroscopy-based techniques, can be considered as alternatives for conventional methods in order to solve food safety-related problems [58]. For instance, applying AI has been rising since 2015, and is expected to stay on the same path for the next 10 years. In fact, combining analytical technologies with AI and data analysis tools, along with developing sensing systems, is expected to have great potential for the agriculture and food industry [64,88]. However, many of these techniques are still at the research and development stages [81], Sensor applications, for example, and developing robust algorithms are still areas for further exploration [64]. It appears that the driving factors to execute more innovation within a certain applied field, and if we are talking about AI in the food industry, are mainly the Industrial Revolution and the need for intelligent systems, hence smart factory development [87]. AI tool applications in the food industry have been intense in 2020 mostly, where researchers were regarded as carrying out more research work using the AI method, which might be linked to the recent pandemic situation due to the COVID-19 virus. Many industrial corporations are now open to AI applications as an alternative for human workers, for cheaper and better outcomes.

There has been much confusion within the literature, especially when it comes to food safety and quality concepts, as well as concerning soilborne and foodborne infections in crop foods. These misconceptions were mostly present in papers classified as analytical techniques or AI-oriented journals. On the other hand, food technology and/or biology-oriented papers tend to the nonadequate referring to AI tools, maybe due to miscommunication between these research areas. Generally speaking, we may suggest that research-wise, this topic might stumble accross these challenges:

- The complexity and diversity of food matrices, which may require different AI models and parameters for different food products and contaminants.
- The lack of standardization and validation of AI methods and data, which may affect the accuracy, reliability, and comparability of the results.
- The potential risks and uncertainties of AI applications in agriculture, such as environmental impacts, socioeconomic impacts, cyber-attacks, biases, and errors, which may affect food security, sustainability, and resilience.
- The need for interdisciplinary collaboration and stakeholder engagement, which may involve challenges such as communication barriers, knowledge gaps, cultural differences, and conflicting interests.

### 5.1. Big Data

**Figure 10:**
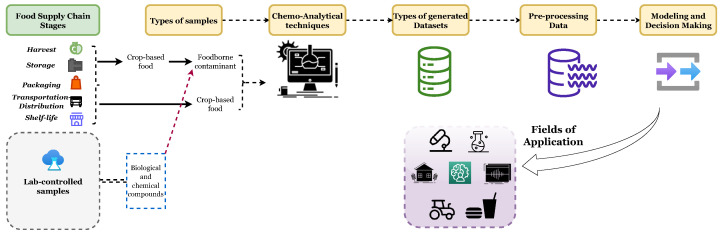
Information and data workflow according to case studies within our collected papers.

The complexity of food matrices that represent samples in experiments (Figure 10) is an important challenge. Their diversity leads to a variety of data types, hence various protocols and methods. Contaminant detection, for example, laboratory detection, emphasizes accuracy, while field detection emphasizes efficiency and portability [81]. Spectral data can be very noisy and susceptible to continuous change [53]. Big data are a major challenge, where food safety relies on data analysis and the processing of information coming from a variety of resources, such as IoT and information-based systems. At the macro level, data analysis impacts market operations and government policies; at the micro level, it is called the smart supervision of food safety. Creating a food traceability system can establish a full correlation between all resources from different stages of the food supply chain. It can be represented as a food safety management platform based on big data, forming a data warehouse, with a unified and robust data management system, for digital food safety supervision [105]. In fact, large data are required to develop cutting-edge technologies, but difficulties arise, such as confusion between data fitting and modeling, and inadequate decision making. Also, there is the curse of dimensionality, data leakage, limited real datasets to experiment on, and low productivity [113].

### 5.2. Learning Methods, XAI, and Interpretability

**Figure 11:**
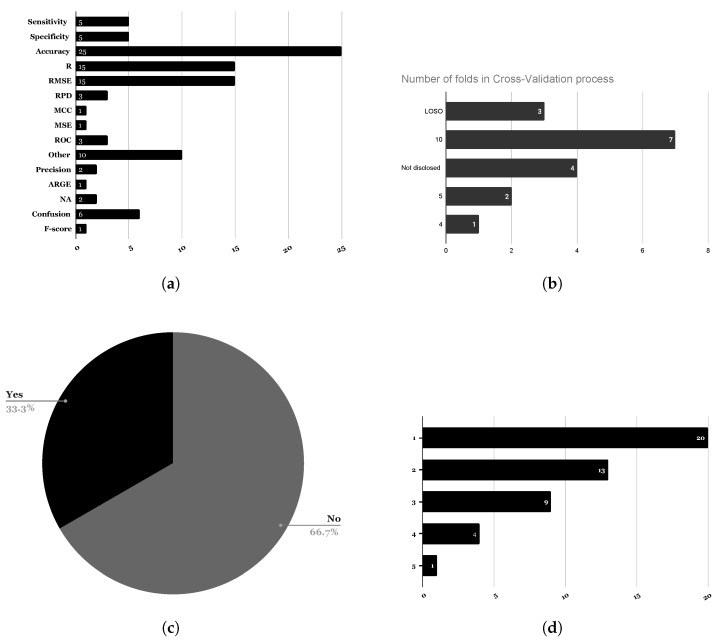
Statistics about evaluation metrics and the use of K-fold cross-validation in the research papers. (**a**) Number of times each evaluation metric is used in the 49 research papers. (**b**) Count of evaluation metrics used per research paper. (**c**) Percentage of research papers that uses K-fold cross-validation for model validation. (**d**) The number of folds used in K-fold.

Limited model performances are also a major issue, along with the need for combining this analytical methods with robust nonlinear algorithms [76]. Also, various general guidelines are in place to objectively assess a machine learning model performance, in particular the use of cross-Validation approaches (also known as K-fold cross-validation) to avoid different problems such as over-fitting. However, as depicted in Figure 11c, only 33% of the article papers used cross-validation techniques, with the number of folds ranging between 4 and 10 (see Figure 11d). Also, the evaluation metrics used to validate the model performance are of upmost importance. As shown in Figure 11a, accuracy is the most used metric. In addition, over 42% of research papers only used one metric and an additional 27% only used two metrics to assess the model performance.

Nondestructive techniques in food safety, especially when it comes to spectral imaging, are the objective detection of defects in plants. However, many challenges arise, such as being restricted to a limited number of attributes; there is a need for more efficient image acquisition and processing, in HSI or NIR, for example. The challenge that remains is that of the standardization of techniques; each reported study is more or less limited to a specific instrumental parameter, studying a single food item and particular defect, while using a different learning algorithm or a different validation process [82]. Limitations and challenges of rapid methods are mainly technical but sometimes are depending on the right choice of data analysis techniques, including the most studied features, without losing information [113]. In most cases of spectral data analysis, it is hard to acknowledge the effect of variable combinations and adding interpretability in decision making.

### 5.3. Real-World Applications

The robustness and transferability of models is one of the main challenges and one of the limitations that restrict the wide-scale application of spectroscopic techniques [69,80,81]. Most of these research papers are only investigations of the feasibility of applications of these approaches. More research should be conducted in routine analysis and real-time implementation [62]. The practical application of emerging techniques, such as the E-nose, still needs to overcome a series of problems, such as the robustness and transitivity of models [81]. Not every mentioned technology seems to be at the same development level in theory as those in laboratories of the food industry; many are still at the infancy level [113], for example, in safety cereal processing, which needs to be further investigated [100]. There should also be more focus on establishing open-source databases and accessible analytical methods for more applications [81].

To our knowledge, there are only a few published papers involving spectral sensing coupled with AI tools, especially for crop-based food safety, although many pieces of research related to food safety and quality, in general, have been executed. Additionally, most studies combing analytical methods with AI tools were from contributors who were mainly scholars of computer sciences and/or related fields, where in-depth safety problem solving is not the main objective of the study, but rather general or specific feature engineering [69,70,80].

## 6. Conclusions and Future Work

This work conducts a qualitative as well a quantitative evaluation of research works regarding our research topic. Furthermore, it proposes a unique methodology to search the literature exhaustively and come up with relevant conclusions and suggestions for further research. This methodology can be further applied in upcoming comprehensive review papers or for searching the literature in emerging areas of research. We executed many searches with many keywords, which was necessary to perform an exhaustive search in the literature to obtain as many papers as possible. Nonetheless, we remarked that most papers were majorly discussing quality assessment issues, mostly, food origins and/or adulteration, using classification and/or regression methods. Also, there was a high tendency to confuse safety and quality measures, which are clearly separate. Since we opted to accept reviews that are remotely related to our specific topic, these review papers were discussing a diversity of points, including our target problem, with more details directed towards meat products, and quality assessment measures, more than safety matters. Food safety, like any other real-life problem, has either been a principal topic in research projects or simply an application to test decision-making tools. It is apparent that there are few applications, and even studies, directed towards food safety when it comes to its contamination prevention, where most studies focused mainly on animal products, like milk and meat; hence, very few studies were regarding crop-based food. To this end, machine learning methods are of crucial importance. Analytical techniques and analytical strategies are completely different concepts: the first one is related to one field of studies involving for example spectral techniques; the second one represents an intersection between many areas of research, hence diverse technologies, as it was discussed and referred to in many case studies papers and review papers, like systems for detecting food defects involving spectral imaging, chemometrics for preprocessing, and computer vision for image analysis and decision making.

There have been many gaps while searching the literature. We at this moment encourage more work to be conducted regarding this problem, for a variety of reasons. First, food safety-security is a global call into question, congregating multiple areas of research and industry. In our study, we investigated crop-food safety while considering all steps of the food supply chain. There are many challenges in optimizing the whole process, where employing AI tools would be highly beneficial. Food contamination prevention is a real-world challenge, with multiple hazards at stake. Our focus was mainly on crop-based foods, regarding the whole process, starting from healthy soil and fresh seeds to grow these crop foods to the marketed food product.

As discussed previously, different contaminants present serious problems, particularly biological contaminants. The first step in ensuring both food security and food safety is growing a healthy crop. On one hand, crop diseases have multiple impacts, decreasing the yield, which leads to insufficient food staples, defecting yield products and causing unsafe food for both humans and animals. Also, regarding farm animals, once they ingest diseased foods, all animal-based foods are susceptible to being unsafe for consumption. Furthermore, there is an apparent gap between AI studies’ development and their applications, not only at the industrial level but at the research level as well, particularly in crop and agricultural sciences. Henceforth, upcoming research should be directed towards developing cutting-edge technologies in precision agriculture, by executing more studies in the fields of crop sciences combined with machine learning and computer vision tools. Our future work will be focusing mainly on solving problems related to inspecting resistant crops to biological contaminants in arid and semiarid regions as an important aspect of food safety. Working on drought-resistant crops and combining AI tools is one of the major challenges facing research globally, and African countries specifically. This problem falls within the area of phytopathology, crop improvement, and machine learning. Our quest is to work on tasks related to crop classification while adding interpretability in deep learning and complex ML techniques based on image processing.

## Figures and Tables

**Table 1:** Latest applications combining AI tools with analytical approaches for food safety and quality.

Analytical Approach	AI Tool	Problematic	Ref.
Spectroscopy	Python-based portable system using Jetson TX2 Module	Food classification of four classes of coffee and purées	[37]
Near-infrared spectroscopy	Block sparse Bayesian learning (BSBL) with fast marginalized likelihood maximization (FMLM)	Computational cost reduction for calculating the inverse of a large matrix containing absorption peak information	[38]
Impedance spectroscopy	A fuzzy logic model applied on the parameters extracted from distribution of relaxation times (DRT)	Meat-based food classification according to its freshness for different types of muscles	[39]
TeraHertz (THz) spectroscopy and chemometrics	Interval partial least squares (iPLS) for optimizing the THz frequency and other preprocessing techniques combined with extreme learning machine (ELM), genetic algorithm support vector machine (GA-SVM), and artificial bee colony algorithm support vector machine (ABC-SVM) for decision making	Three typical soybean origins’ identification	[40]
Fourier transform infrared (FTIR) spectroscopy	FTIR and multispectral imaging (MSI) coupled with support vector machines (SVM) for regression	Meat quality assessment, specifically minced pork patties stored under modified atmosphere packaging (MAP) conditions, by estimating the microbial population	[41]
Raman spectroscopy	A single convolutional neural network (CNN) model development where hyperparameters, activity functions, and loss functions were optimized	Spectral data preprocessing simplification	[42]
Dielectric spectroscopy	Principal component analysis (PCA) for preprocessing and four models, namely support vector machine—SVM, K-nearest neighbor—KNN, linear discriminant—LD and quadratic discriminant—QD, for classification purposes	Discrimination between three citrus juices in order to develop new technologies to identify adulteration	[43]

**Table 2:** Search strings with the highest number of results (>30) for Web of Science and IEEE Xplore.

IEEE Xplore	WOS
“neural networks” + “spectroscopy” + “food”: **63**	“Machine learning” + “spectroscopy” + “food”: **235**
“neural networks” + “chemical analysis” + “food”: **58**	“neural networks” + “spectroscopy” + “food”: **160**
“Artificial intelligence” + “chemical analysis” + “food”: **57**	“computer vision” + “spectroscopy” + “food”: **111**
“Machine learning” + “spectroscopy” + “food”: **55**	“Deep learning” + “spectroscopy” + “food”: **77**
“Artificial intelligence” + “spectroscopy” + “food”: **50**	“Machine learning” + “spectroscopy” + “crop”: **69**
“Machine learning” + “chemical analysis” + “food”: **43**	“Artificial intelligence” + “Analytical strategies” + “food”: **37**

**Table 3:** This table represents all collected 69 papers, where A stands for Articles and R stands for Review papers.

Title	First Author	Publisher	Type	Year	Ref.
Detection of defects on selected apple cultivars using hyperspectral and multispectral image analysis	Mehl	ASABE	A	2001	[47]
Differentiation and detection of microorganisms using Fourier Transform infrared photoacoustic spectroscopy	Irudayaraj	Elsevier	A	2002	[48]
Rapid detection of foodborne microorganisms on food surface using Fourier transform Raman spectroscopy	Yang	Elsevier	A	2003	[49]
Differentiation of food pathogens using FTIR and artificial neural networks	Gupta	ASABE	A	2005	[50]
Identification and quantification of foodborne pathogens in different food matrices using FTIR spectroscopy and artificial neural networks	Gupta	ASABE	A	2006	[51]
Applications of Artificial Neural Networks (ANNs) in Food Science	Huang	Taylor & Francis	R	2007	[52]
Supervised pattern recognition in food analysis	Berrueta	Elsevier	R	2007	[53]
Cortical Networks Grown on Microelectrode Arrays as a Biosensor for Botulinum Toxin	Scarlatos	Wiley-Blackwell	A	2008	[54]
Detecting single Bacillus spores by surface enhanced Raman spectroscopy	He	Springer	A	2008	[55]
Self-organizing algorithm for classification of packaged fresh vegetable potentially contaminated with foodborne pathogens	Siripatrawan	Elsevier	A	2008	[56]
Raman Spectroscopy-Compatible Inactivation Method for Pathogenic Endospores	Stöckel	American society for Microbiology	A	2010	[57]
Application of Hyperspectral Imaging in Food Safety Inspection and Control: A Review	Feng	Taylor & Francis	R	2012	[58]
Characterization of food spoilage fungi by FTIR spectroscopy	Shapaval	Wiley-Blackwell	A	2013	[59]
Detection of aflatoxin contaminated figs using Near-Infrared (NIR) reflectance spectroscopy	Güneş	IEEE	A	2013	[60]
Hyperspectral and multispectral imaging for evaluating food safety and quality	Qin	Elsevier	R	2013	[61]
Analytical techniques combined with chemometrics for authentication and determination of contaminants in condiments: A review	Reinholds	Elsevier	R	2015	[62]
Applications of computer vision for assessing quality of agri-food products: a review of recent research advances	Ma	Taylor & Francis	R	2016	[63]
Data mining derived from food analyses using non-invasive/non- destructive analytical techniques; determination of food authenticity, quality & safety in tandem with computer science disciplines	Ropodi	Elsevier	R	2016	[64]
Image analysis operations applied to hyperspectral images for non-invasive sensing of food quality—A comprehensive review	ElMasry	Science Direct	R	2016	[65]
Early Warning Modeling and Application based on Analytic Hierarchy Process Integrated Extreme Learning Machine	Geng	IEEE	A	2017	[66]
Feasibility of Non-Destructive Internal Quality Analysis of Pears by Using Near-Infrared Diffuse Reflectance Spectroscopy	Shen	IEEE	A	2017	[67]
FT-IR Hyperspectral Imaging and Artificial Neural Network Analysis for Rapid Identification of Pathogenic Bacteria	Lasch	American Chemical Society	A	2018	[68]
An Approach for the Development of a Sensing System to Monitor Contamination in Stored Grain	Kaushik	IEEE	A	2019	[69]
Application of Deep Learning in Food: A Review	Zhou	Wiley-Blackwell	R	2019	[70]
Machine learning algorithms for the automated classification of contaminated maize at regulatory limits via infrared attenuated total reflection spectroscopy	Öner	Wageningen Academic publishers	A	2019	[71]
Quantitative assessment of zearalenone in maize using multivariate algorithms coupled to Raman spectroscopy	Guo	Elsevier	A	2019	[72]
Raman Spectroscopy Classification of Foodborne Pathogenic Bacteria Based on PCA-Stacking Model	Wan-dan	IEEE	A	2019	[73]
Rapid determination of aflatoxin B1 concentration in soybean oil using terahertz spectroscopy with chemometric methods	Liu	Elsevier	A	2019	[74]
Terahertz Spectroscopy Determination of Benzoic Acid Additive in Wheat Flour by Machine Learning	Sun	Springer	A	2019	[75]
An Overview on the Applications of Typical Non-linear Algorithms Coupled With NIR Spectroscopy in Food Analysis	Zareef	Springer	R	2020	[76]
Application of deep learning and near infrared spectroscopy in cereal analysis	Le	Elsevier	A	2020	[77]
Arcobacter Identification and Species Determination Using Raman Spectroscopy Combined with Neural Networks	Wang	American Society for Microbiology	A	2020	[78]
Deep learning networks for the recognition and quantitation of surface-enhanced Raman Spectroscopy	Weng	The Royal Society of Chemistry	A	2020	[79]
Development of Machine Learning & Edge IoT Based Non-destructive Food Quality Monitoring System using Raspberry Pi	Sahu	IEEE	A	2020	[80]
Emerging techniques for determining the quality and safety of tea products: A review	Yu	Wiley-Blackwell	R	2020	[81]
Machine learning applications to non-destructive defect detection in horticultural products	Nturambirwe	Science Direct	R	2020	[82]
Multi-view Learning for Subsurface Defect Detection in Composite Products: a Challenge on Thermographic Data Analysis	Wu	IEEE	A	2020	[83]
On-line prediction of hazardous fungal contamination in stored maize by integrating Vis/NIR spectroscopy and computer vision	Shen	Elsevier	A	2020	[84]
Optical detection of aflatoxins B in grained almonds using fluorescence spectroscopy and machine learning algorithms	Bertania	Elsevier	A	2020	[85]
Achieving a robust Vis/NIR model for microbial contamination detection of Persian leek by spectral analysis based on genetic, iPLS algorithms and VIP scores	Rahi	Elsevier	A	2021	[86]
Application of Artificial Intelligence in Food Industry—a Guideline	Mavani	Springer	R	2021	[87]
Applications of THz Spectral Imaging in the Detection of Agricultural Products	Ge	MDPI	R	2021	[88]
Bioimpedance data statistical modelling for food quality classification and prediction	Rivola	IEEE	R	2021	[89]
Characterisation and Classification of Foodborne Bacteria Using Reflectance FTIR Microscopic Imaging	Xu	MDPI	A	2021	[90]
Combining optical spectroscopy and machine learning to improve food classification	Magnus	Elsevier	A	2021	[91]
Deep Learning for Rapid Identification of Microbes Using Metabolomics Profiles	Wang	MDPI	A	2021	[92]
Hyperspectral image processing for the identification and quantification of lentiviral particles in fluid samples	Gómez-González	Nature Publishing Group	A	2021	[93]
Identification of the apple spoilage causative fungi and prediction of the spoilage degree using electronic nose	Guo	Wiley-Blackwell	A	2021	[94]
Investigation of nonlinear relationship of surface enhanced Raman scattering signal for robust prediction of thiabendazole in apple	Li	Elsevier	A	2021	[95]
Metaheuristic Optimization to Improve Machine Learning in Raman Spectroscopic based Detection of Foodborne Pathogens	Vakilian	IEEE	A	2021	[96]
Microwave Sensing for Food Safety: a Neural Network Implementation	Ricci	IEEE	A	2021	[97]
Non-destructive detection of foreign contaminants in toast bread with near infrared spectroscopy and computer vision techniques	Yin	Springer	A	2021	[98]
Raman spectroscopy combined with machine learning for rapid detection of food-borne pathogens at the single-cell level	Yan	Elsevier	A	2021	[99]
Recent advances in assessing qualitative and quantitative aspects of cereals using nondestructive techniques: A review	Zareef	Elsevier	R	2021	[100]
Trace Identification and Visualization of Multiple Benzimidazole Pesticide Residues on Toona sinensis Leaves Using Terahertz Imaging Combined with Deep Learning	Nie	MDPI	A	2021	[101]
A Novel Method for Carbendazim High-Sensitivity Detection Based on the Combination of Metamaterial Sensor and Machine Learning	Yang	MDPI	A	2022	[102]
Advances in Machine Learning and Hyperspectral Imaging in the Food Supply Chain	Kang	Springer	R	2022	[103]
Component spectra extraction and quantitative analysis for preservative mixtures by combining terahertz spectroscopy and machine learning	Yan	Elsevier	A	2022	[104]
Design of Food Safety Supervision System in the Background of Big Data	Zhang	IEEE	R	2022	[105]
Detection and quantification of peanut contamination in garlic powder using NIR sensors and machine learning	Rady	Academic Press Inc.	A	2022	[106]
Domain Adaptation for In-Line Allergen Classification of Agri-Food Powders Using Near-Infrared Spectroscopy	Bowler	MDPI	A	2022	[107]
Investigation of reflectance, fluorescence, and Raman hyperspectral imaging techniques for rapid detection of aflatoxins in ground maize	Kim	Elsevier	A	2022	[108]
Low-Resolution Raman Spectroscopy for the detection of contaminant species in algal bioreactors	Adejimi	Elsevier	A	2022	[109]
Machine learning-based typing of Salmonella enterica O-serogroups by the Fourier-Transform Infrared (FTIR) Spectroscopy-based IR Biotyper system	Cordovana	Elsevier	A	2022	[110]
Markov Transition Field Combined with Convolutional Neural Network Improved the Predictive Performance of Near-Infrared Spectroscopy Models for Determination of Aflatoxin B1 in Maize	Wang	MDPI	A	2022	[111]
Potential application of hyperspectral imaging in food grain quality inspection, evaluation and control during bulk storage	Aviara	Elsevier	R	2022	[112]
Recent Advances and Applications of Rapid Microbial Assessment from a Food Safety Perspective	Pampoukis	MDPI	R	2022	[113]
Recent Progress in Spectroscopic Methods for the Detection of Foodborne Pathogenic Bacteria	Hussain	MDPI	R	2022	[114]
Spectroscopy and imaging technologies coupled with machine learning for the assessment of the microbiological spoilage associated to ready-to-eat leafy vegetables	Manthou	Elsevier	A	2022	[115]

**Table 4:** Chemometrics and ML approaches for crop-based food safety and quality in some relevant review papers.

Ref.	Crop-Food	Preprocessing Steps	Decision Model
[81]	Tea	Standard normal variate (SNV) and multiplicative scatter corrections	PCA, KNN, KPCA, ANN, HCA, BPNN, PLS, CPNN, SPA, PNN, ELM, LDA, SVM, S-LDA, LVQ, KLDA, MLP, RBF, RF
[103]	Apple, wolfberry, lettuce, pear, green plum, peach, strawberry, Brassica, jujube, lettuce and chives	LLE, LE, ISOMAP and MDS, SNE and t-SNE	PLSR, KNN, LDA, NB, DT, SVR, SVM, RF, LSSVM, LWR, FNN, ResNet, CNN, and DNN
[76]	Grain products, forages, oil, fruits, vegetables, sugarcane seeds, coffee, tea, spices, black/green tea, grapes, apples, wheat flour, rice and barley	Multivariate calibration of spectral data, standard normal variate transformation (SNV), multiplicative scatter correction (MSC), smoothing, derivative, wavelet transforms (WT), and orthogonal signal correction (OSC)	ANN, BP-ANN, GA-ANN, RBFNN, AdaBoost, SVM, LA, ELM, SLFN, LS-SVR, SVM, linear, radial basis function (RBF), normalized polynomial, sigmoid, Gaussian RBF, and string kernels
[62]	Condiments, spices, and herbs	PCA, HCA, parallel factor analysis (PARAFAC), MPLS, PLS, ANOVA, t-test, straight line subtraction (SLS), constant offset elimination (COE), and minimum–maximum normalization (MMN)	ANN, kNN, PLS regression, PLS-DA, HCA, PCA, LDA, k-means cluster analysis (KM-CA), and DA
[87]	Fruits	computer vision systems	Adaptive Neuro Fuzzy Inference System (ANFIS)
[70]	Vegetables	CNN	ResNet-152, AlexNet-SVM classifier, and hybrid CNN-SSAE
[58]	Apples, cucumbers, spinach, and wheat	PCA, PLSR	PLSDA, ANN, LD, and PCA

## Data Availability

Data is contained within the article.
